# Professional Standing

**Published:** 1895-10

**Authors:** 


### EDITORIAL.

### Professional Standing.

A recent number of the *Lancet-Clinic* has an editorial on this subject that contains thoughts and suggestions pertinent to the physician, and not only to him, but to any who are engaged in any line of practice of the healing art—the Dentist, for instance.

The writer gives expression to the following: "All physicians have many interests in common, and these interests are of such a nature as to be justifiably likened to family relationships. If one is injured, all suffer; if one prospers, all rejoice. * * * * A suit for malpractice on the part of a known and reputable practioner cannot be successfully prosecuted without considering the question as to who are well known and reputable practitioners; the answer to this may be found by a reference to the roll of the county or local medical society, for it is in the home organization or family circle that a man's true character and ability are best known. Hence the great advantages that accrue to the physicians by their being identified in a favorable way with their immediate neighbors. The county society is in fact a guarantee and a protective association for those who belong to it. The men who are not identified with their home society have no reason to believe that they have any professional standing whatever, which is usually true. Nearly all the professional work of physicians is done single-handed and alone. In that way an admirable individuality is built up, but sooner or later there comes to every one a period when association is necessary; when this time does come to a man, who loved and attended his county society, supporting friends will come to him from every direction.

In the home organization all members have common ties and common interests. The man whose name is not found on the society list has something the matter with his professional escutcheon—something wrong with his degree; he has a suspicious, or actually bad, professional record, with whom a consultation is a hazardous risk.

The man who is conscious that he is all right, has a recognition of his claim from his reputable neighbors. On the other hand, the man who is conscious that there exists a blur on his record, keeps it out of sight, and himself under cover, so far as he may.

It is a good thing for physicians and especially young men to become identified with their county societies."

The principles involved in the above statements are as applicable to the dentist as to the general physician. Dentists are engaged in a common cause, all have the same difficulties to meet and to overcome, and ought to have common sympathies, and every true professional dentist will be a help and support to his fellows. In order to secure the best results in this direction, friendship should be cultivated and maintained; societies should everywhere be organized and sustained and all truly professional dentists should be interested and actively engaged in such work; it is helpful to the individual, and to his confreres as well, and above all secures the greatest benefit to those whom it is the business of a dentist to serve; and in this direction there is the greatest need, there are none who do not more or less need help in this respect. Every one might be more efficient if he would, and verily there is need.

Association and co-operation is the order of the day in almost every human occupation. In many departments of far less importance and value to humanity than dentistry is, there far more perfect, extensive and efficient organization.

Let us all ponder these facts, and each appreciate his responsibility, and act in obedience to his convictions.

